# Known unknowns and unknown unknowns in suicide risk assessment: evidence from meta-analyses of aleatory and epistemic uncertainty

**DOI:** 10.1192/pb.bp.116.054940

**Published:** 2017-06

**Authors:** Matthew Large, Cherrie Galletly, Nicholas Myles, Christopher James Ryan, Hannah Myles

**Affiliations:** 1 University of New South Wales, Australia; 2 University of Adelaide, Australia; 3 Royal Adelaide Hospital, Australia; 4 University of Sydney, Australia

## Abstract

Suicide risk assessment aims to reduce uncertainty in order to focus treatment and supervision on those who are judged to be more likely to die by suicide. In this article we consider recent meta-analytic research that highlights the difference between uncertainty about suicide due to chance factors (aleatory uncertainty) and uncertainty that results from lack of knowledge (epistemic uncertainty). We conclude that much of the uncertainty about suicide is aleatory rather than epistemic, and discuss the implications for clinicians.

Uncertainty is the psychological state of being unsure, of having doubt, of not fully knowing. Uncertainty is central to modern medicine, where its recognition drives diagnostic efforts and leads to the pursuit of evidence-based practice. All medical decision-making occurs under conditions of varying uncertainty about diagnosis, optimal treatment and prognosis. This is true in the assessment of suicidal patients.

Uncertainty has two underlying components: epistemic uncertainty that results from a lack of knowledge, and aleatory uncertainty that results from random or chance events.^1–3^ In medical practice, both types of uncertainty are at play. A teenage tobacco user might or might not develop cancer later in life. This is mostly a matter of chance, a chance that will increase with heavier and longer tobacco use. This longitudinal cancer risk is probabilistic, akin to the throw of a die, and further knowledge might not greatly reduce uncertainty about what will eventually happen. In middle age, the same smoker might develop haemoptysis. A chest X-ray would reduce uncertainty about the presence or absence of lung cancer, but it might be more clearly resolved by a biopsy. Uncertainty in this case is not probabilistic – the smoker either has or does not have cancer. This is now a question that can be resolved with more information. Chance is no longer playing a part.

It is generally considered that uncertainty in suicide risk assessment can be greatly reduced by a detailed assessment of the patient's suicidal thoughts, plans and actions, and attention to other demographic and clinical factors. Suicide risk assessment guidelines and relevant peer-reviewed publications often contain long lists of questions to ask and factors to consider.^4–7^ This approach assumes that more substantial knowledge of the patient, their illness, circumstances and intentions will reduce the epistemic uncertainty in the assessment. Few would doubt that chance also plays a major part in suicide. The course of underlying illness, the vagaries of individual decision-making and impulsivity, and the patient's future circumstances are all sources of aleatory uncertainty.

In this article we consider the uncertainty surrounding suicide using the framework of epistemic and aleatory uncertainty. In order to do this, we use recent meta-analytic research to interrogate the proposition that uncertainty about suicide risk can be reduced by knowing more about suicidal thoughts and behaviours, or by the knowledge of a wider range of suicide risk factors.

## Can knowing about suicidality reduce uncertainty about suicide?

No small number of references could begin to do justice to the importance that suicidal ideation and behaviours have assumed in suicide research. Several recent systematic meta-analyses have synthesised the quantitative peer-reviewed literature on the statistical relationship between suicidality and suicide. Each of these meta-analyses has cast doubt on the notion that knowing more about suicide ideas, or suicidality more broadly, reduces uncertainty about suicide.

Two meta-analyses published in 2011, one examining risk factors for suicide by psychiatric in-patients^8^ and the other examining risk factors for suicide by recently discharged patients,^9^ found that the association between suicidal ideation and suicide was statistically weak, with diagnostic odds ratios (OR) of less than 3. In 2015, Chapman *et al*^10^ published a meta-analysis finding that suicidal ideation was significantly associated with suicide among patients with schizophrenia spectrum conditions. However, suicidal ideation was not significantly more likely to lead to suicide than no suicidal ideation among patients with mood disorders (OR = 1.49, 95% CI 0.92–2.42).

A 2016 meta-analysis^11^ examined the broader question of whether self-injurious thoughts and behaviours deserve their status as strong predictors of future suicidal behaviour. This study found that self-injurious thoughts and behaviours are only weakly associated with later suicide attempts (OR = 2.14, 95% CI 2.00–2.30) and death from suicide (OR = 1.54, 95% CI 1.39–1.71). The authors concluded that assessments of suicidality provided an improvement in prognostic accuracy that was only marginally above chance.

Another 2016 meta-analysis examined the psychometric properties of both individual risk factors and suicide risk assessment scales (the Beck Hopelessness Scale, Suicide Intent Scale and Scale for Suicide Ideation) among populations of people who self-harm.^12^ The authors found a modest statistical association between previous self-harm and suicidal intent and later suicide, concluding that individual risk factors are ‘unlikely to be of much practical use because they are comparatively common in clinical populations’. With respect to use of suicide risk scales they considered that they ‘may provide false reassurance and [are], therefore, potentially dangerous’.

Thus, five recent meta-analytic summaries of the peer-reviewed literature have each reached similar conclusions – knowing about suicide thoughts and behaviours can only reduce uncertainty about future suicide to a modest extent.

## Can knowing about a wider range of risk factors reduce uncertainty about suicide?

If enquiries about our patients' suicide ideas, plans and actions do not help very much, what else should mental health professionals do to reduce uncertainty? The most common and obvious answer is to consider a comprehensive range of other suicide risk factors. Again it is simply not possible to describe the full range of articles, guidelines and peer-reviewed papers that consider the range of potentially important risk factors for suicide. A weakness of this literature is that although very large numbers of risk factors for suicide have been identified, there is no widely accepted way in which this information can be combined to improve the predictive strength of suicide risk assessment. Further, despite widespread recommendations for a comprehensive consideration of suicide risk factors, there are doubts as to whether combining risk factors can ever produce clinically useful predictive models. More than 30 years ago, Pokorny^13^ concluded his paper describing a landmark prospective suicide prediction study with the statement that it ‘is inescapable that we do not possess any item of information or any combination of items that permit us to identify to a useful degree the particular persons who will commit suicide, in spite of the fact that we do have scores of items available, each of which is significantly related to suicide’.

We recently published a meta-analysis that further examined the dilemma posed by Pokorny.^14^ We synthesised the results of all the published longitudinal prospective studies that used multiple risk factors to model future suicide among cohorts of psychiatric patients. We included experimental studies that employed multiple regression or survival analysis and studies that validated suicide risk prediction instruments. Our main outcome measure was the odds of suicide in high-risk patients compared with lower-risk patients. One of the aims of the meta-analysis was to determine if the observed between-study variability in this OR could be explained by the number of risk factors used in the predictive modelling. The results were very clear. We found a pooled OR of 4.84 (95% CI 3.79–6.20) derived from 37 studies and 53 samples of patients. This indicates that the rate of suicide among high-risk patients can be expected to be about 5 times the rate of suicide of low-risk patients. While this sounds like it might be a clinically useful finding, these odds do not meaningfully improve on the pooled ORs of about 4 that are associated with some individual suicide risk factors among psychiatric patients – factors such as depression, hopelessness and prior suicide attempts.^8,9^ The meta-analysis also found that 56% of suicides occurred in high-risk groups (sensitivity) and 44% occurred among the lower-risk group. Over an average follow-up of 5 years, 5.5% of high-risk patients, but 1% of low-risk patients, died by suicide. This 5.5% suicide mortality over a period of 5 years means the probability of suicide of high-risk patients over clinically important durations is extremely low. For example, the weekly probability of suicide of a high-risk patient over the 5-year follow-up can be estimated at 0.055/(5×52) = 0.0002115 or 1 in 4700 people. In practical terms, what this means is that if a patient is deemed at higher risk of suicide because of the presence of one or more risk factors (recall that the number of risk factors seems unimportant), our best estimate of the incidence of suicide in the following week is about 1 in 4700. Even if there was a hypothetical dynamic risk factor that transiently increased the next-week risk of suicide by 10 times, strict supervision of almost 500 high-risk people for 1 week would be needed to prevent one suicide – assuming that such supervision were 100% effective.

**Fig. 1:**
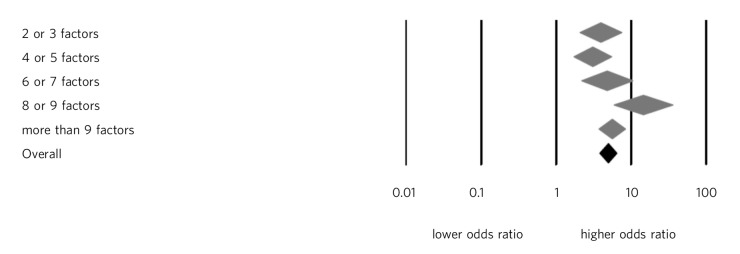
Odds ratios of the predictive strength of multivariate suicide risk assessment according to the number of factors in the predictive model. Diamonds indicate the pooled estimate and the (overlapping) 95% confidence intervals. Data from Large *et al*.^14^

Relevant to the present paper, the meta-analysis found that the predictive models that used more suicide risk factors had no more statistical strength, and no better discrimination between high-risk and lower-risk groups, than studies that used fewer factors (slope 0.007, 95% CI−0.016 to 0.03, *P* = 0.53). In fact, studies that employed two factors had a similar predictive strength to studies that employed ten or more factors. Figure 1 plots the diagnostic odds with 95% confidence intervals effect size of models using 2 or 3 factors (8 samples), 4 or 5 factors (11 samples), 6 or 7 factors (7 samples), 8 or 9 factors (5 samples) and 10 or more factors (22 samples) with obviously overlapping confidence intervals. We concluded that multivariate models offered little advantage over single risk factors and that multivariate models that relied on more suicide risk factors performed no better than those that use fewer risk factors.

## Implications of the limits to epistemic uncertainty

The findings of these recent meta-analytic studies undermine one of our profession's main assumptions about suicide risk assessment. Suicidal ideation,^10^ suicide behaviour^11,12^ and more complex modelling^14^ offer predictive advantages only a little better discrimination than chance. Hence, most of our uncertainty about suicide risk is aleatory; knowing more does not help because epistemic uncertainty plays only a minor part.

So what should clinicians do? First, we believe that this fundamental problem with suicide risk assessment needs to be acknowledged. We need to acknowledge our powerlessness to usefully classify individuals or groups of patients according to future suicide risk. We need to acknowledge this to ourselves, and communicate this to health departments, to the courts, and most importantly, to our patients and their families.

Second, we need to provide a more universal standard of care, involving a complete and sympathetic assessment of every patient, their illness and their circumstances. Such assessment is needed to guide individualised treatment plans, and might also have the intrinsic benefit of reducing suicidality.^15^ Where modifiable risk factors are found, we need to try to modify them. For example, patients who present with suicidal ideation when intoxicated should not be summarily discharged when sober and denying suicidal ideation, but should be offered access to addiction services that have some prospect of reducing suicide risk and improving their lives, irrespective of their overall risk category.

Third, we need to be very sparing in our use of involuntary treatment as a reaction to suicide risk. It is likely that very few patients who we admit to hospital would have died by suicide as out-patients over the period of time usually associated with a contemporary length of stay. Patients making ongoing immediate threats might still be admitted to hospital, as such threats are a crucial communication and legitimate focus of care without recourse to notions of probability. However, suicide risk is simply not a sufficient warrant for making paternalistic decisions about involuntary hospital care. Equally, we should be careful not to automatically deny low-risk patients voluntary in-patient treatment when they want it. Many suicides are by low-risk patients and we should not pretend we are able to peer into their future any more than we can discern the future of a higher-risk patient.
